# Deep Learning to Estimate Left Ventricular Ejection Fraction From Routine Coronary Angiographic Images

**DOI:** 10.1016/j.jacadv.2023.100632

**Published:** 2023-10-11

**Authors:** Behrouz Rostami, Kenneth Fetterly, Zachi Attia, Apurva Challa, Francisco Lopez-Jimenez, Jeremy Thaden, Samuel Asirvatham, Paul Friedman, Rajiv Gulati, Mohamad Alkhouli

**Affiliations:** Department of Cardiovascular Medicine, Mayo Clinic, Rochester, Minnesota, USA

**Keywords:** artificial intelligence, coronary angiography, deep learning, left ventricular ejection fraction

## Abstract

**Background:**

Cine images during coronary angiography contain a wealth of information besides the assessment of coronary stenosis. We hypothesized that deep learning (DL) can discern moderate-severe left ventricular dysfunction among patients undergoing coronary angiography.

**Objectives:**

The purpose of this study was to assess the ability of machine learning models in estimating left ventricular ejection fraction (LVEF) from routine coronary angiographic images.

**Methods:**

We developed a combined 3D-convolutional neural network (CNN) and transformer to estimate LVEF for diagnostic coronary angiograms of the left coronary artery (LCA). Two angiograms, left anterior oblique (LAO)-caudal and right anterior oblique (RAO)-cranial projections, were fed into the model simultaneously. The model classified LVEF as significantly reduced (LVEF ≤40%) vs normal or mildly reduced (LVEF>40%). Echocardiogram performed within 30 days served as the gold standard for LVEF.

**Results:**

A collection of 18,809 angiograms from 17,346 patients from Mayo Clinic were included (mean age 67.29; 35% women). Each patient appeared only in the training (70%), validation (10%), or testing set (20%). The model exhibited excellent performance (area under the receiver operator curve [AUC] 0.87; sensitivity 0.77; specificity 0.80) in the training set. The model’s performance exceeded human expert assessment (AUC, sensitivity, and specificity of 0.86, 0.76, and 0.77, respectively) vs (AUC, sensitivity, and specificity of 0.76-0.77, 0.50-0.44, and 0.90-0.93, respectively). In additional sensitivity analyses, combining the LAO and RAO views yielded a higher AUC, sensitivity, and specificity than utilizing either LAO or RAO individually. The original model combining CNN and transformer was superior to DL models using either 3D-CNN or transformers.

**Conclusions:**

A novel DL algorithm demonstrated rapid and accurate assessment of LVEF from routine coronary angiography. The algorithm can be used to support clinical decision-making and form the foundation for future models that could extract meaningful data from routine angiography studies.

Cine angiography is the cornerstone of invasive cardiology practice. Images obtained from standard angiographic projections are utilized to determine the presence, extent, and characteristics of coronary disease. Like other imaging modalities, images obtained via cine angiography contain a wealth of ‘other’ data besides information about luminal stenosis.^1^, ^2^, ^3^ This includes possible information about ventricular contractile function, valvular and extracardiac calcifications, lung capacity, and diaphragmatic movement.^4^ However, noncoronary findings on cine angiography are usually discarded. This is due to the limited available data and the challenges of focusing on noncoronary findings in real time while evaluating coronary disease during routine patient care.

In recent years, deep learning (DL) algorithms have found extensive application for image and video classification across various domains including medical imaging.^5^, ^6^, ^7^, ^8^ The automatic hierarchical feature extraction capability of CCNs allows them to discover intricate relationships between input and output.^9^ Additionally, a family of a novel DL framework called transformer has demonstrated exceptional performance in image and video analysis. Transformers utilize the Attention module in their architecture, enabling them to consider both local and global information while processing data from lower to higher layers.^10^, ^11^, ^12^

Recent advances in artificial intelligence provide an opportunity to extract additional information from data routinely acquired in the cath lab. Howard et al showed that DL can facilitate automatic detection of guide catheter dampening while recording aortic pressure waveforms and the prediction of catheter-induced coronary injuries.^13^^,^^14^ There are only a few studies to date that leverage routine coronary angiography images to screen for myocardial dysfunction.^15^ Therefore, we sought to address this knowledge gap using a large dataset of consecutive patients referred to the Cardiac Catheterization Laboratory at Mayo Clinic. We hypothesized that DL algorithms can readily predict with high accuracy the presence of moderate-severe left ventricular (LV) dysfunction among patients undergoing coronary angiography. We proposed that such a model can be incorporated into the routine catheterization laboratory’s workflows, allowing inexpensive real-time identification of LV dysfunction without the need for LV angiography.

## Methods

### Study Cohort

Consecutive adult patients undergoing coronary angiography at Mayo Clinic sites in Minnesota, Florida, and Arizona between January 1, 2015, and December 31, 2021, was included. This study was conducted after the approval of the institutional review board at Mayo Clinic. Patients who did not consent to have their medical records used for research were excluded.

### Pre-processing

Two left coronary angiograms per study were selected for DL assessment. For each patient, we selected the left anterior oblique (LAO) caudal (20°-60°/10°-40°) and right anterior oblique (RAO) cranial (0°-40°/15°-45°) X-ray projections to be processed for the assessment of ventricular function. We excluded cases with low number of frames, those with missing angle values, and those with missing patient identification. No patient was excluded based on stenosis percentage or patient/table movement. In our practice, angiogram images are acquired at a frame rate of 15 frames per second. Due to computer hardware limitations and a need to minimize processing times, a limited number of frames were extracted and forwarded to the DL model. First, frames 1 to 15 (1 second), which correspond to LCA filling with iodine-based contrast, were discarded. Next, 16 frames per cine image were extracted starting from the 16th frame and by selecting every other frame and stored in .png format. The dataset forwarded to the DL algorithm corresponded to the 2-second interval ranging from seconds 2 to 4 of the coronary angiogram with 7.5 fps sampling rate. After frame extraction, LCA videos were identified using a custom, DL-based, LCA vs right coronary angiogram classifier. Finally, the LCA data samples were randomly split into train, validation, and test sets with the portions of 70%, 10%, and 20% of the total number of samples, respectively.

### Model architecture

**Figure 1:**
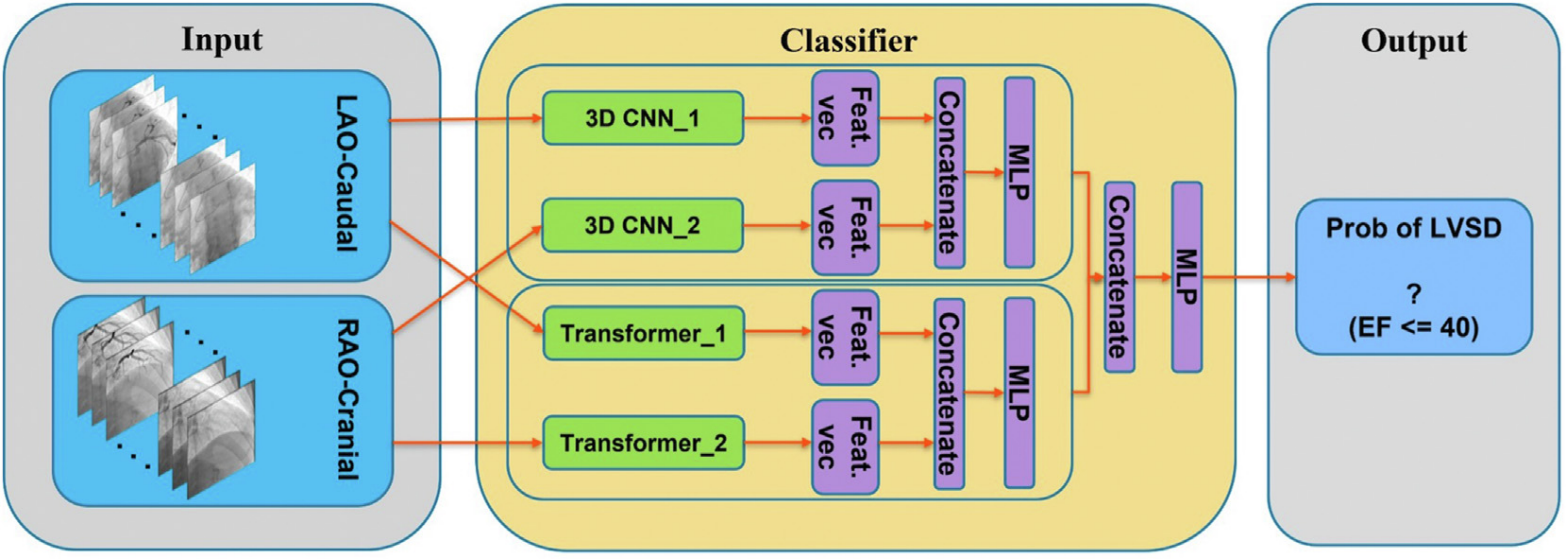
Flow Chart and Model Architecture CNN = convolutional neural networks; EF = ejection fraction; LAO = left anterior oblique; LVSD = left ventricular systolic dysfunction; MLP = multilayer perceptron; RAO = right anterior oblique.

**Figure 2:**
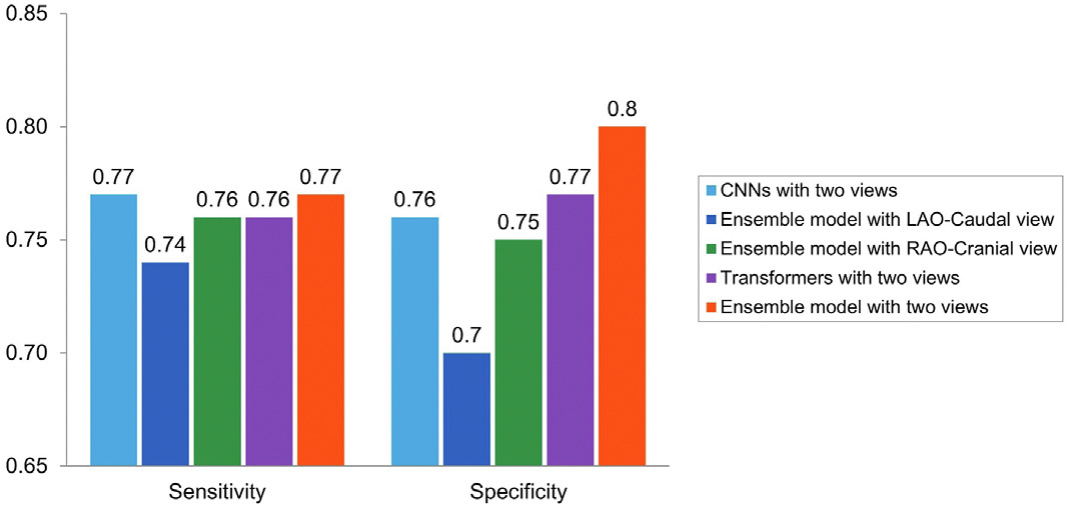
Performance of the CNN, Transformer, and Ensemble Models CNN = convolutional neural network; LAO = left anterior oblique; RAO = right anterior oblique.

The DL architecture used was an ensemble model that takes the advantage of combining 3D convolutional neural networks (CNNs) and transformers in parallel (Figure 1). Figure 2. The 3D-CNN architecture is a 3D version of the ResNet model, which is widely used for video classification.^16^ The ResNet solves some of the common issues connected with other architectures including the vanishing gradient problem.^17^ There are several versions of ResNet architecture with different numbers of layers including ResNet18, ResNet34, ResNet50, etc. In this study, the architecture with 152 layers, ResNet152, was utilized. In addition to the 3D CNN, a transformer architecture, TimeSformer, that was proposed in 2021 for video classification tasks was also implemented.^18^

During the training phase, for each study, 2 cine images were fed into separate 3D CNNs (Figure 1), while there was no weight sharing between them. The output of the 2 networks would be concatenated to create a new feature vector, which was fed into a simple multilayer perceptron (MLP) with a single hidden layer to generate the final output from the 3D CNN part. All 3D CNNs and the MLP model parameters were optimized simultaneously. The same approach was followed using 2 separate transformers to obtain the transformer part output. At the final stage of the classifier, the outputs of 3D CNNs and the transformers were concatenated and fed into a simple MLP to generate the final ensemble classifier’s outcome. The final model’s output is the predicted label, which is either positive (left ventricular ejection fraction [LVEF] ≤40%) or negative (LVEF >40%). The ground truth (labels) obtained based on the patients’ LVEF as measured on a transthoracic echocardiogram done within 30 days in Mayo Clinic Echo Labs. The hypothesis was that the combination of the 3D-CNN and the transformer models would boost the overall performance. Class weights were assigned to the loss function to handle the imbalanced data issue. All the models were implemented using the Python programming language (version 3.7.11) and in PyTorch (version 1.7.1) framework.

### Model evaluation

After the training phase, the trained model was applied on the test set to evaluate the performance. The classification threshold value obtained from the validation samples was utilized during the test phase to predict the labels. Multiple measurements were used to evaluate the model’s performance including area under the receiver operator curve (AUC), sensitivity, and specificity.^19^ To further evaluate the model’s performance, the model output was compared to expert human performance on an additional test set. A subset of 290 angiograms (not included in the DL model angiogram) was randomly selected. Two experienced board-certified interventional cardiologists from Mayo Clinic’s cath lab provided their prediction on the LVEF of the studies by a visual assessment of 2 views of each angiogram (LAO and RAO). Cardiologists carefully examined the videos and estimated the LVEF, considering the artery displacement and heart motion speed. The human performance was then compared with the DL output. Both cardiologists were blinded to the DL model’s and each other’s predictions.

#### Sensitivity analyses

We performed additional analyses first using single angiographic projections (either LAO or RAO) in isolation to ascertain how each individual angiographic projection contributed to the final output, and second using one of the DL methods in isolation (either CNN or transformer) to discern the incremental value of combining the 2 algorithms.

## Results

A total of 27,290 angiogram studies (n = 23,273) were initially obtained from the Mayo Clinic cath lab. After applying the exclusion criteria, 18,809 studies from 17,346 patients were included for model training (70%; 13,157 cases from 12,142 patients), validation (10%; 1,890 cases from 1,734 patients), and testing (20%; 3,762 cases from 3,470 patients). Of these, 3,354 cases from 3,240 patients had an abnormal LVEF value (EF ≤40%). Number of abnormal cases/patients in each of the train, validation, and test sets are 2,353/2,273, 336/327, and 665/640, respectively. Mean age was 67.29 years, and 35% were women. A total of 8,582 patients (49.5%) presented with acute myocardial infarction as the indication for the coronary angiogram.

**Figure 3:**
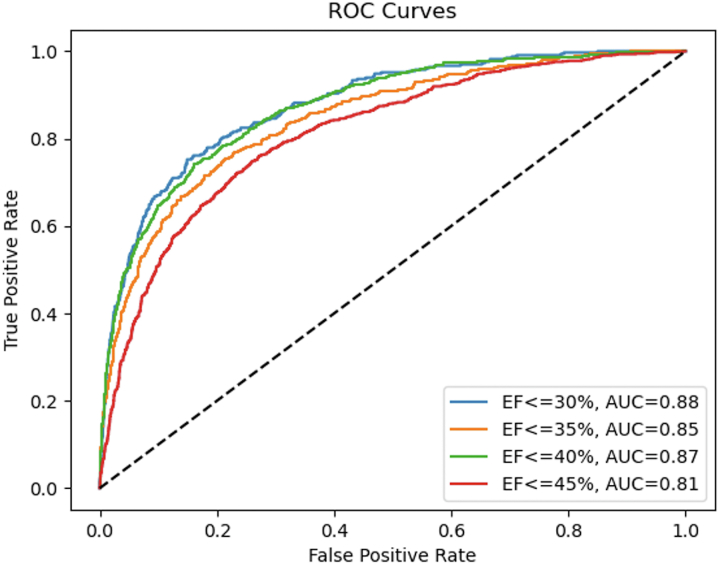
Performance of the Ensemble Deep Learning Model for Various Left Ventricular EF Thresholds AI = artificial intelligence; AUC = area under the curve; EF = ejection fraction.

**Figure 4:**
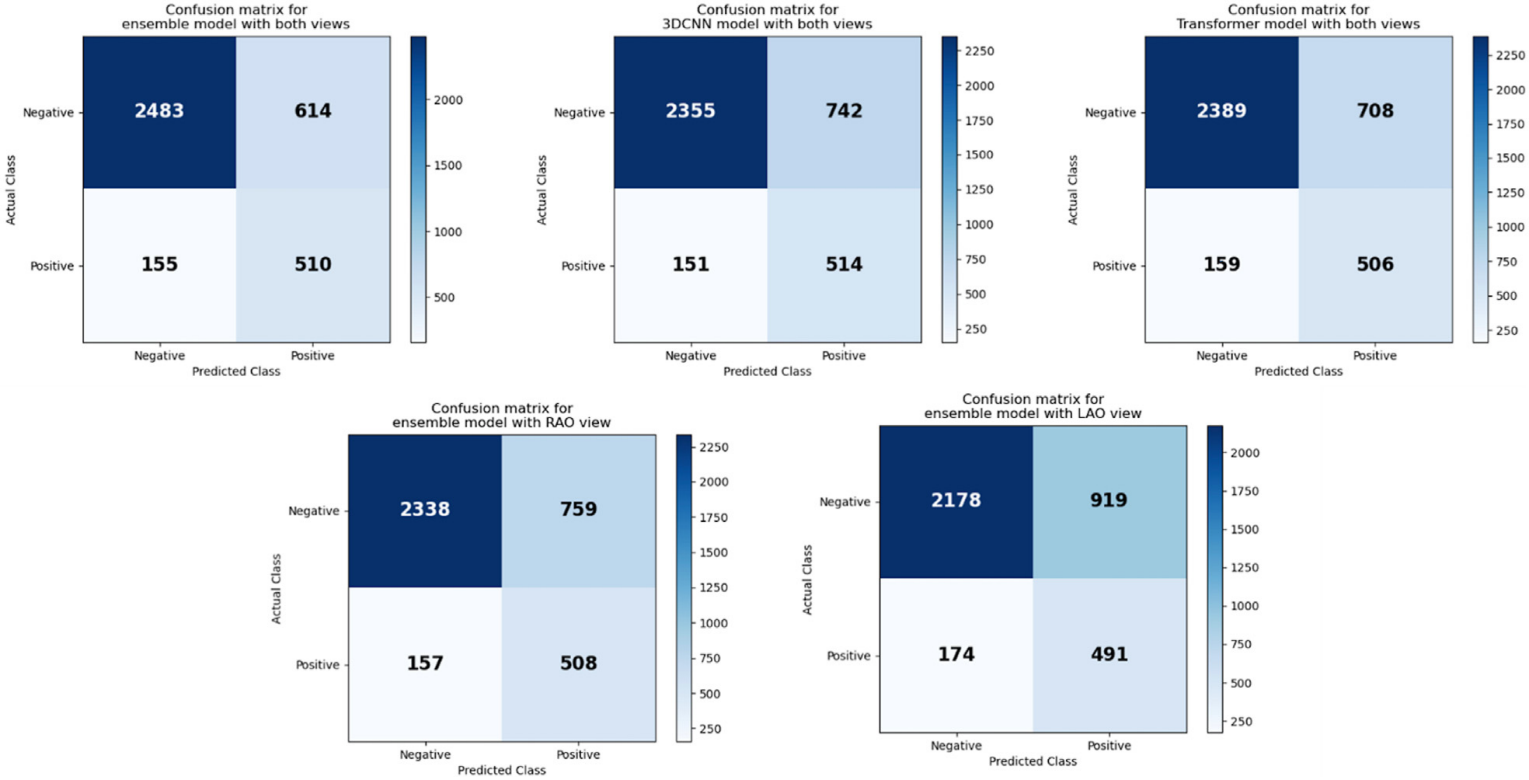
Confusion Matrices for the CNN, Transformer, and the Ensemble Models Abbreviations as in Figure 1.

The training time was 15 hours for 25 epochs, while the inference time was recorded as 22 minutes on the test set population. When the trained model was applied on the testing set, the AUC, sensitivity, and specificity were 0.87, 0.77, and 0.80, respectively. To understand how each of the views contributes to the outcome individually, the cine images from single views were fed into the model in separate experiments as well. Using only LAO-caudal view, the model achieved an AUC of 0.80, a sensitivity of 0.74, and a specificity of 0.70. Using only RAO-caudal view, the model achieved an AUC of 0.84, a sensitivity of 0.77, and a specificity of 0.75. Also, when only 3D CNNs were used (without combining with transformers) and both views were utilized as the inputs, the AUC of 0.85, sensitivity of 0.77, and specificity of 0.76 were achieved, while using only the transformer part resulted in 0.84, 0.76, and 0.77 values for these parameters (Supplemental Methods). Additionally, Figure 3 illustrates the model’s performance with different LVEF thresholds, and Figure 4 shows the confusion metrics for each of the artificial intelligence models utilized.

**Figure 5:**
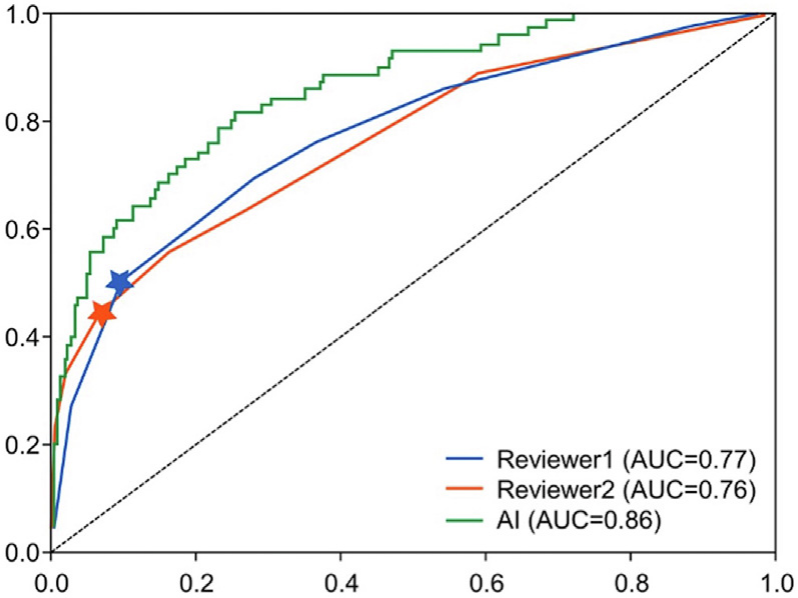
Performance of the Combined Ensemble Deep Learning Model vs Expert Human Assessment AUC = area under the curve.

**Figure 6:**
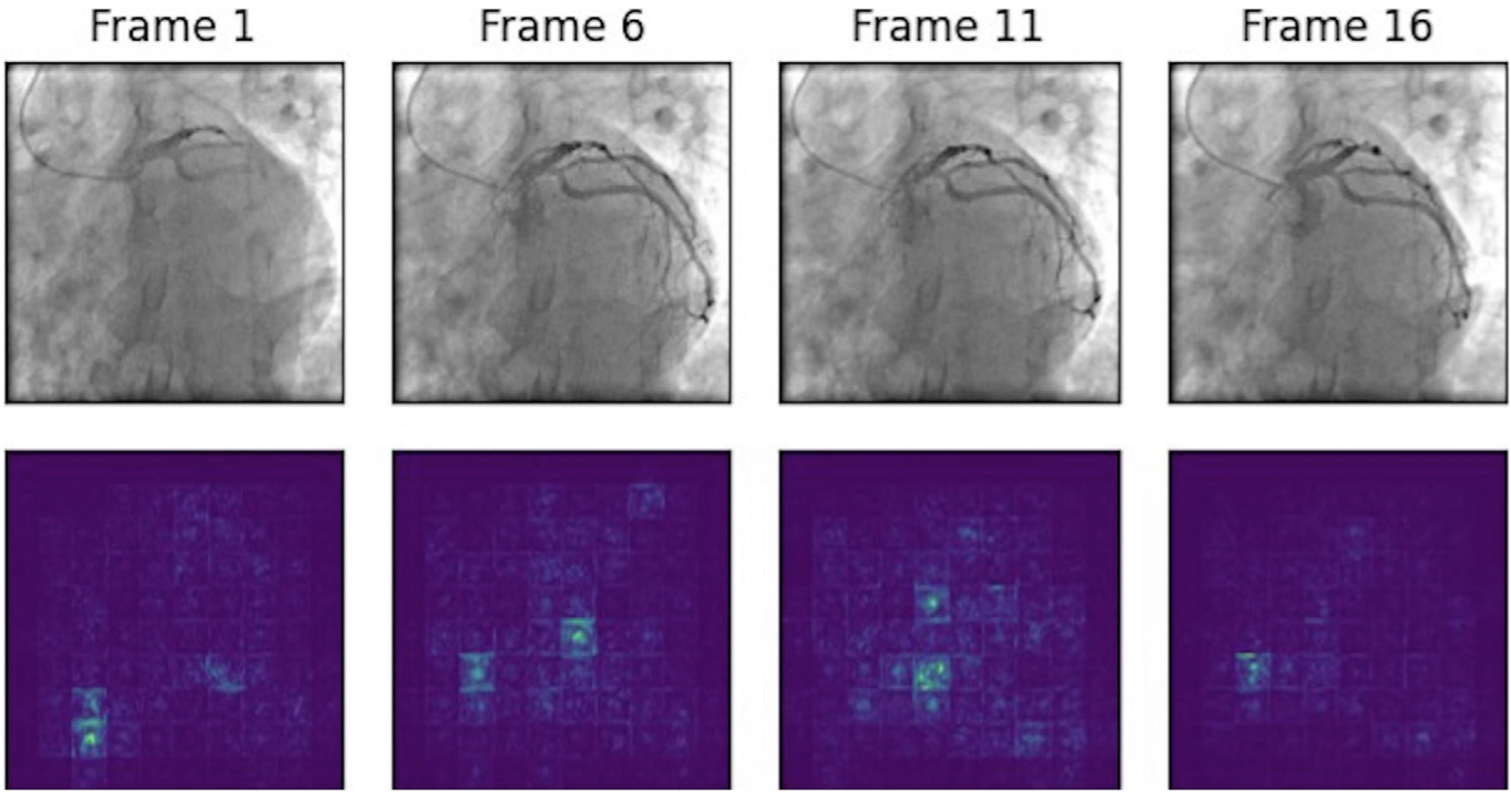
Sample Left Anterior Oblique Caudal Frames With Their Corresponding Saliency Maps

**Figure 7:**
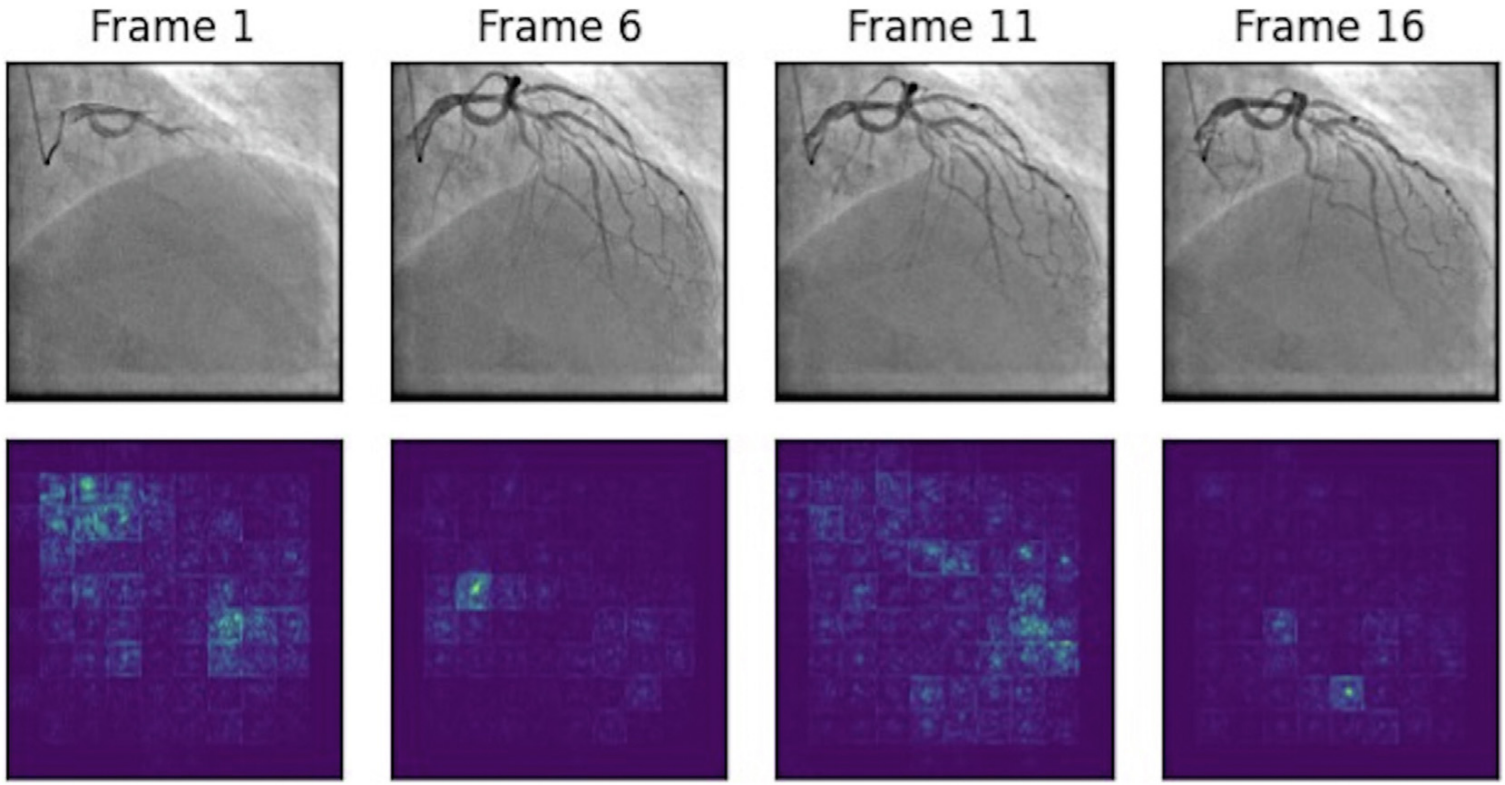
Sample Right Anterior Oblique Cranial Frames With Their Corresponding Saliency Maps

The performance of the artificial intelligence ensemble model was compared to human performance by applying the model on an additional test set. The DL-based algorithm generated superior outcomes when compared to human performance. The human performance was reported with AUCs of 0.76 to 0.77, sensitivity of 0.50 to 0.44, and specificity of 0.90 to 0.93 in comparison with the ensemble model’s AUC of 0.86, sensitivity of 0.75, and specificity of 0.77 (Figures 5). Figures 6 and 7 illustrate the saliency maps from sample frames obtained in the LAO and ROA projections, respectively.

## Discussion

In this study, we examined the feasibility of extracting noncoronary data from routine angiograms using DL. Our hypothesis was that the minute displacement of the epicardial coronary arteries during myocardial contraction could be captured on fluoroscopy and processed with DL algorithms to infer LV contractile function. Our study confirmed the hypothesis and documented that a novel DL method combining 3D-CNN and transformers can discern reduced LVEF from 2 angiographic projects (Central Illustration). These findings have important clinical and investigational implications that deserve further discussion.

**Central Illustration:**
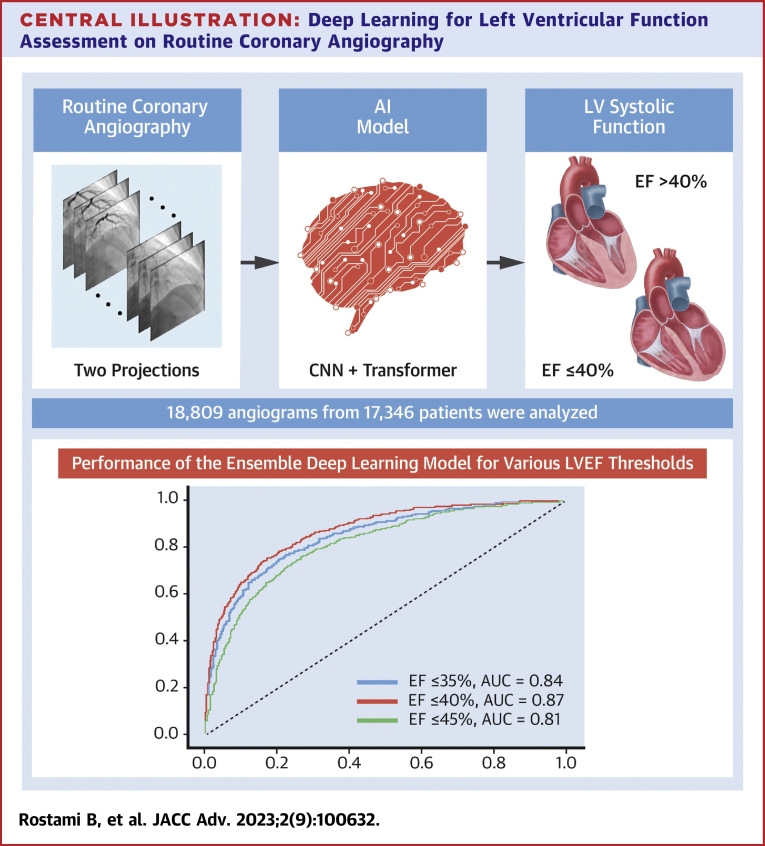
Deep Learning for Left Ventricular Function Assessment on Routine Coronary Angiography Using 2 orthogonal views (LAO caudal and RAO cranial) from >17,000 patients who had recent echocardiographic assessment of LV function, a novel deep learning model incorporating convolutional neural network and transformer approaches to identify patients with reduced LV ejection fraction. The models had the best performance (AUC 0.87) for classifying LVEF as >40% or ≤40%. AUC = area under the curve; CNN = convolutional neural networks; EF = ejection fraction; LAO = left anterior oblique; LVEF = left ventricular ejection fraction; RAO = right anterior oblique.

Clinically, many patients present urgently to the cath lab with no prior echocardiographic examination. Often, those patients require complex high-risk coronary interventions. Knowledge of their LVEF could aid the interventionalist in real-time assessment of the risks and benefits of the intervention. For instance, an interventionalist planning to proceed with urgent stenting of the left main or proximal left anterior descending coronary artery might alter the interventional approach (ie, consider mechanical circulatory support) if real-time assessment of ventricular function suggests the presence of a very low LVEF. Furthermore, this DL method is inexpensive, simple, and could reduce the need for LV angiography and additional iodine contrast usage. Once externality is validated, the model can then be incorporated into the workflow in the lab by automatically displaying the predicted LVEF value on the screen after 2 angiographic images from 2 orthogonal projections are completed.

In addition to the clinical value that can be realized at present, the study has several connotations with regards to future investigations of DL in the catheterization laboratory: First, the study documents the incremental value of merging 2 angiographic projections and combining 2 different artificial intelligence architectures (3D CNN and transformer) to maximize the model’s predictive performance. Albeit intuitive, the integration of different images and different DL algorithm to boost DL algorithms in the cath lab has not previously been described. Also, the Delong test results provided evidence to support the hypothesis that the performance of the ensemble model was superior when compared to either the standalone 3DCNNs or the transformers. Second, the study’s approach can be applied to cine images for the prediction of other important clinical factors such as the presence of right ventricular dysfunction, pulmonary hypertension, severe valve disease, or volume overload. Currently, the ability of DL to discern these parameters has been limited to echocardiography and electrocardiography.^20^, ^21^, ^22^ Third, the ability of DL to discern local displacement of coronary arteries can be further developed to study other features of the coronary angiogram itself that are understudied. For example, DL could quantify vessel tortuosity and assess its association with device success or clinical outcomes. Similarly, DL could also potentially assess the impact of ectasia or aneurysmal coronary disease on outcomes.

### Study Limitations

First, for the purpose of this exploratory study, we dichotomized the LVEF values into ≤40% vs >40%. In clinical practice, it is more meaningful to provide an actual estimated valve of the LVEF. However, conducting such a study would require a much larger sample of angiogram and computational capabilities that are not available at present. This will be the focus of further collaborative multicenter studies. Second, the data were all derived from Mayo Clinic catheterization laboratories, which allow technical consistency but preclude the ability to externally validate the study’s findings. Third, while the AUC achieved with this approach is excellent (0.87), it is lower than the AUC reported in other DL studies in other areas of cardiology (eg, predicting low LVEF or silent atrial fibrillation from electrocardiograms).^23^^,^^24^ This could be related to the complex nature of analyzing moving vs static images. However, collaborative studies using various DL techniques could potentially further improve the model’s performance. Fourth, the interpretability of the model is an issue that should be investigated more in the future studies. As can be found in Figures 6 and 7, part of the extracted features obtained by the transformer stream of the model are clinically meaningful, but certain features still lack apparent clinical relevance, which is related to the common black box issue in DL models. This lack of transparency underscores the need for further research to enhance interpretability and improve the reliability of the model.

## Conclusions

A novel DL approach combining CNN and transformers readily allows assessment of LV contractile function in the cath lab using routine cine angiography images. The algorithm could aid clinical decision-making in real-time and could potentially be modified to extract additional meaningful data from routine angiographic studies.

PERSPECTIVES **COMPETENCY IN PATIENT CARE AND PROCEDURAL SKILLS:** Cine angiography images contain a wealth of data beyond information on vessel stenosis. We demonstrated the feasibility of estimating LVEF with machine learning using routine coronary angiograms.

**TRANSLATIONAL OUTLOOK:** Operators could assess left ventricular function in real time in the catheterization laboratory. Future models can also be developed to obtain other meaningful data from the angiogram.

## Funding support and author disclosures

The authors have reported that they have no relationships relevant to the contents of this paper to disclose.
