# Supplementary material for: Deep Learning to Estimate Left Ventricular Ejection Fraction From Routine Coronary Angiographic Images
Source: JACC Adv. 2023 Oct 11;2(9):100632. doi: 10.1016/j.jacadv.2023.100632 (PMC11198437; doi:10.1016/j.jacadv.2023.100632)
Supplement: Supplementary data [file mmc1.docx]

**SUPPLEMENTAL APPENDIX**

**Supplemental Methods**

**LCA/RCA classifier**

For classifying the angiograms into LCA and RCA groups, we used a 3D Resnet architecture with 152 layers (ResNet-152). The model showed a high-level performance with the test set AUC of 0.967.

**Model development:**

Regarding that the deep learning ensemble models have been used successfully in image and video analysis applications in different fields within the recent years (1-3), we utilized an ensemble model in our study to end up with a superior performance. The ensemble models take the advantage of combining two or more models to provide a performance which is superior to each of the individual models (3).

The ResNet152 architecture is one of the state-of-the-art deep CNNs that showed a high performance in different video classification applications in the literature and then we selected it as one of the models in our research. Also, TimeSformer was the first Transformer-based architecture that was proposed for analyzing videos and generated high performance along with simplicity in implementation and strength in capturing time domain dependencies in videos. As the two architectures use different feature extraction strategies, we hypothesized that they would cover each other weakness when being combined that will improve the overall performance, something that was confirmed based on our experiments.

For each of the CNN or Transformer stream of our ensemble model we used separate models for each of the two videos which didn’t share weights. We hypothesized that when two separate models are used, they would be able to focus on features which are specific to that view (projection) that will improve the performance in comparison with the case that only a single model is used which shares the weights. The hypothesis is that in the latter case, the model would focus more on the features which are in common between the two views which results in a lower performance. This hypothesis was confirmed during our experiments.

For training the models we used two GPUs (Nvidia Tesla V100-PCIE) with Adam optimizer along with the learning rate of 1e-5, batch size of 32 (16 for the Transformers), and 25 as the maximum number of epochs.

For handling the imbalanced dataset issue, we trained the models with and without using the weight-assigning strategy during loss calculation and the results were similar. The idea behind the weight-assigning is to consider a higher weight for the class with less samples to increase their contribution in loss calculation and use lower weights for the majority class. Different weight assigning strategies including inverse proportion of class frequencies were tried in this study.

**Optimum classification threshold**

The optimum classification threshold obtained during the training phase where we applied the trained model on validation set and calculated the optimum threshold based on having a tradeoff between True Positive Rate (TPR) and False Positive Rate (FPR). In fact, the optimum point is the point on the ROC curve that is the closest one to the point with the coordinate (0,1) on the diagram which corresponds to the top left corner. This point can be obtained by finding the threshold value and then the TPR and FPR values which make this distance, sqrt((1-TPR)^2 + FPR^2), minimum.


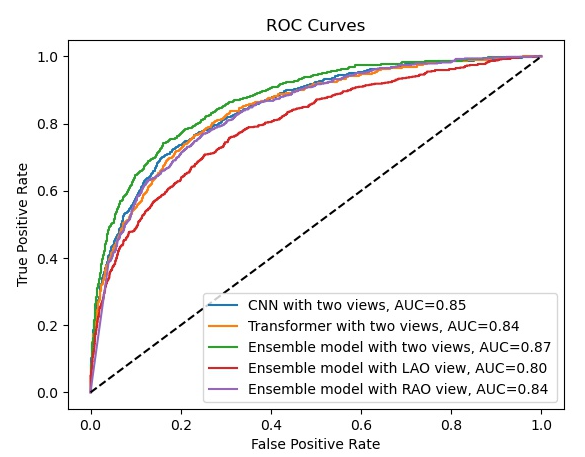


**Comparing performance of different models**

In the main text it was mentioned that for comparison, we also trained the Transformer and 3D CNN architectures separately using two views as input, as well as training the ensemble model using individual views. The ROC curves along with the corresponding AUC values related to these models can be found in Figure 8.

**References**

1. Ganaie MA, Hu M, Malik A, Tanveer M, Suganthan P. Ensemble deep learning: A review. Engineering Applications of Artificial Intelligence 2022;115:105151.

2. Zheng J, Cao X, Zhang B, Zhen X, Su X. Deep ensemble machine for video classification. IEEE transactions on neural networks and learning systems 2018;30:553-565.

3. Rostami B, Anisuzzaman D, Wang C, Gopalakrishnan S, Niezgoda J, Yu Z. Multiclass wound image classification using an ensemble deep CNN-based classifier. Computers in Biology and Medicine 2021;134:104536.
